# Impaired Albumin Uptake and Processing Promote Albuminuria in OVE26 Diabetic Mice

**DOI:** 10.1155/2016/8749417

**Published:** 2016-10-16

**Authors:** Y. S. Long, S. Zheng, P. M. Kralik, F. W. Benz, P. N. Epstein

**Affiliations:** ^1^Department of Pediatrics, University of Louisville, Louisville, KY, USA; ^2^Department of Pharmacology and Toxicology, University of Louisville, Louisville, KY, USA

## Abstract

The importance of proximal tubules dysfunction to diabetic albuminuria is uncertain. OVE26 mice have the most severe albuminuria of all diabetic mouse models but it is not known if impaired tubule uptake and processing are contributing factors. In the current study fluorescent albumin was used to follow the fate of albumin in OVE26 and normal mice. Compared to normal urine, OVE26 urine contained at least 23 times more intact fluorescent albumin but only 3-fold more 70 kD fluorescent dextran. This indicated that a function other than size selective glomerular sieving contributed to OVE26 albuminuria. Imaging of albumin was similar in normal and diabetic tubules for 3 hrs after injection. However 3 days after injection a subset of OVE26 tubules retained strong albumin fluorescence, which was never observed in normal mice. OVE26 tubules with prolonged retention of injected albumin lost the capacity to take up albumin and there was a significant correlation between tubules unable to eliminate fluorescent albumin and total albuminuria. TUNEL staining revealed a 76-fold increase in cell death in OVE26 tubules that retained fluorescent albumin. These results indicate that failure to process and dispose of internalized albumin leads to impaired albumin uptake, increased albuminuria, and tubule cell apoptosis.

## 1. Introduction

Diabetic nephropathy (DN) is the most common cause of end-stage renal disease [1]. Albuminuria is a primary characteristic of DN and a significant predictor for progression towards renal failure [2]. In addition to serving as a marker, albuminuria contributes to the pathology of DN [3]. Controlling the upward progression of albuminuria is a therapeutic goal for preventing decline in renal function [4] of diabetic patients.

Both glomerular protein leakage and impaired tubular protein uptake can contribute to albuminuria. In healthy individuals urine albumin is maintained at low levels by the minimal amount of protein that passes the glomerular filtration barrier and by tubular reabsorption of protein that does pass the glomerular filtration barrier. Extensive literature from humans and animal models supports a role for a defective glomerular filtration barrier in the development of albuminuria [5–7]. There is also evidence that defective protein reabsorption by proximal tubules plays a significant role [8, 9]. However the significance of impaired tubular reuptake and the experimental evidence for this defect is controversial [10]. One recent mouse study demonstrated albuminuria despite the fact that tubular albumin uptake was higher than normal [11].

The diabetic mouse model which manifests the most profound albuminuria is the OVE26 (OVE) transgenic mouse [12]. Their severe albuminuria is valuable for modeling aspects of advanced human DN and for probing the mechanisms of albuminuria. In this report we provide evidence that the high level of albuminuria in OVE mice is due to glomerular leakage combined with reduced uptake in a subset of tubules. Impaired tubular uptake, severe albuminuria, and increased tubule cell death appear to be secondary to their inability to process and eliminate internalized albumin.

## 2. Methods

### 2.1. Experimental Animals

OVE diabetic mice on the FVB strain background and FVB controls at 4.5–7 months of age were bred in our laboratory. All mice had free access to standard chow and water. Procedures were followed per the guidelines of the NIH Guide for the Care and Use of Laboratory Animals and approved by the University of Louisville Institutional Animal Care and Use Committee.

### 2.2. Urinary Albumin Excretion (UAE)

Individual mice were placed in metabolic cages for 24 hours with access to chow and 10% liquid diet (Glucerna, Abbott Laboratories), as previously described [12, 13]. Urinary albumin was determined using a mouse albumin ELISA kit (Bethyl Laboratories).

### 2.3. Albumin and Dextran Injections and Quantitation

Texas Red and fluorescein-conjugated bovine albumin (TR-albumin and FITC-albumin, resp.) and 70 kDa fluorescein-conjugated dextran (FITC-dextran) were obtained from Invitrogen. The purities of the commercial TR-albumin and FITC-dextran were indicated by the fact that over 98 percent of the fluorescence of the compounds received from the company eluted as a single peak close to bovine serum albumin (BSA) on Sephadex G-75 gel filtration columns (described below). Mice were injected with either TR-albumin, FITC-albumin, or FITC-dextran via the tail vein with a dose of 20 *μ*g/gm body weight. No change in activity was evident in mice for up to four days following albumin or dextran injection. Urine and serum TR-albumin or FITC-dextran were quantified by measuring fluorescence in a Hitachi FU2500 fluorometer at 596/620 nm for TR-albumin or 494/521 nm for FITC. Specimens from noninjected mice of the same type were used to subtract background fluorescence. Sample values were interpolated against known standards of TR-albumin and FITC-dextran. To determine the size of fluorescent fragments in urine, the urine samples were separated into high (>5000 kDa) and low molecular weight components by fractionation on NAP-5 (GE Healthcare) columns. Ten urine samples were separated at higher resolution on an 11.5 cm × 1.5 cm Sephadex G-75 column at a flow rate of 5 mL/hr.

### 2.4. Histology and Immunohistochemistry

Kidneys were fixed overnight in 4% formalin. Paraffin sections were used for visualization of TR-albumin and FITC-dextran or for immunohistochemistry. For immunofluorescence, sections were blocked with normal serum and incubated overnight at 4°C with goat anti-mouse albumin (1 : 600 dilution, Bethyl Laboratories), rabbit anti-lamp1 (1 : 200 dilution, Abcam), or goat anti-megalin (1 : 50 dilution, Santa Cruz). After three washes in PBS, sections were incubated with secondary antibodies for 1 hour at room temperature, followed by an additional three washes. Donkey secondary antibodies (Jackson ImmunoResearch Laboratories) were used at 1 : 50 or 1 : 100 dilution. Images were captured using a Nikon DS-Fi1 camera system with a Nikon E600 microscope.

### 2.5. TUNEL Staining

Kidney paraffin sections were double stained using mouse albumin antibody and the TUNEL staining kit from Chemicon. Briefly, deparaffinized sections were treated with 10 mM sodium citrate pH 5.0 at 50°C before incubation in Chemicon kit equilibration buffer. This was followed by 60-minute incubation at 37°C with the Chemicon kit terminal deoxynucleotidyl transferase enzyme and reaction buffer. Samples were then incubated with Chemicon kit anti-digoxigenin-FITC conjugate for 1 hr at 22°C. Slides were then stained with goat anti-mouse albumin and AMCA conjugated secondary antibody as described in the preceding paragraph. Sections were observed under a 40x objective by FITC fluorescence for TUNEL stained nuclei in tubule cells. Merged images were made for all FITC positive TUNEL nuclei with albumin staining by AMCA blue fluorescence. From pictures of merged image, counts were made of TUNEL stained nuclei in albumin positive and albumin negative tubule profiles. The number of albumin positive tubule profiles was counted directly over the entire kidney section. The number of albumin negative tubule profiles was estimated by counting the average number of negative tubules in 3 random cortical areas per section and then extrapolating this value to the total cortical area of the section. These values were determined from six different OVE mice and were used to calculate the percentage of albumin positive and albumin negative tubule profiles which contained TUNEL positive tubule cells.

### 2.6. Statistical Analyses

Data are expressed as means ± SE. Comparisons between two groups were performed by *t*-test. Correlation was computed as the Pearson product moment correlation coefficient. Statistical analyses were performed with SigmaStat (Systat Software).

## 3. Results

### 3.1. Urine Excretion of Injected Fluorescent Albumin or Dextran

**Figure 1:**
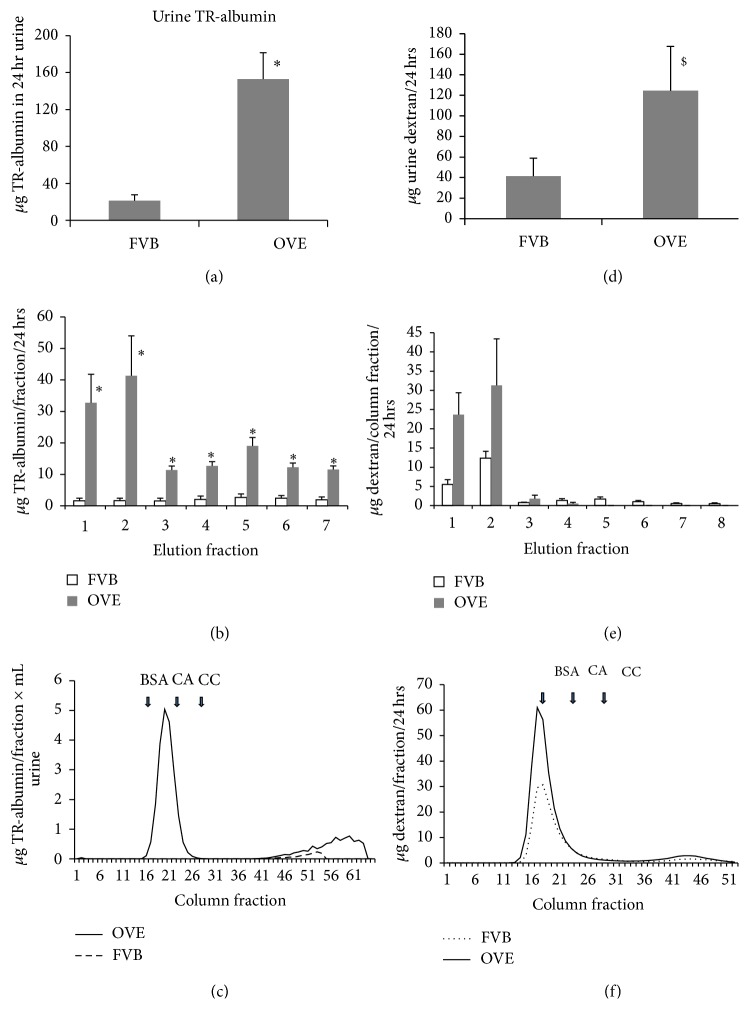
Urine excretion of injected TR-albumin and FITC-dextran. (a) Twenty-four hours after TR-albumin injection, urine samples of OVE mice contain 7-fold more fluorescence than FVB urine. (b) After NAP5 spin column fractionation the first two high molecular weight fractions had 23-fold more fluorescence in OVE urine than in FVB urine. (c) G75 column fractionation profiles of urine show that the high molecular weight peak of OVE urine elutes with the same peak as standard BSA. No TR-albumin peak at this MW could be seen in FVB urine. (d) Twenty-four hours after FITC-dextran injection, urine samples of OVE mice contain 3-fold more fluorescence than FVB urine. (e) After NAP5 spin column fractionation the first two high molecular weight fractions from OVE urine had 3-fold more FITC fluorescence than from FVB urine. (f) G75 column fractionation profiles of urine show that the high molecular weight peak elutes 2 fractions before the standard BSA peak. Arrows indicate peaks for bovine serum albumin (BSA, 66 kDa), carbonic anhydrase (CA, 30 kDa), and cytochrome C (CC, 12 kDa). For panels (a) and (b) results are the average from 4 OVE and 5 FVB mice. Panel (c) results are typical of 3 OVE and 3 FVB mice. Panels (d) and (e) results are the average from 5 OVE and 5 FVB mice. Panel (f) results are typical of 2 OVE and 2 FVB mice. Symbols indicate that OVE values are greater than FVB (^*∗*^ *P* ≤ 0.02, ^#^ *P* ≤ 0.05, and $ indicates a trend of *P* ≤ 0.07).

Mice were injected with fluorescent Texas Red tagged bovine albumin (TR-albumin). The serum level of TR-albumin was measured in 3 OVE and 3 FVB mice at 10 minutes and 24 hours after injection and found to be similar in the diabetic and normal groups (OVE 14.4 ± 0.6 *μ*g/mL versus FVB 15.9 ± 2.9 *μ*g/mL at 10 minutes and OVE 2.3 ± 0.3 *μ*g/mL versus FVB 2.5 ± 0.8 *μ*g/mL at 24 hours). During the 24-hour period following injection OVE mice excreted more TR-fluorescent tag in their urine than FVB mice (Figure 1(a)). Fractionation (Figure 1(b)) on NAP5 columns revealed that OVE urine contained a 23-fold higher content of high molecular weight TR-albumin, in the first two elution fractions, than was present in FVB urine. Fractionation at higher resolution on Sephadex G75 columns (Figure 1(c)) showed that almost all TR-albumin fluorescence in OVE urine eluted at the position of bovine albumin standard (BSA) and that no TR-albumin eluted near this position in FVB urine. All of the TR-albumin from the manufacturer eluted at the position of BSA (not shown) indicating that small fragments of injected TR-albumin are produced by degradation in mice. Since no full length TR-albumin was detectable by high resolution G75 columns, the 23-fold estimate of increased OVE leakage calculated using NAP5 column data is a minimal estimate. These results also demonstrate that Texas Red tagged bovine albumin behaves similarly to what we reported [12] for endogenous mouse albumin with respect to severe albuminuria in OVE diabetic mice.

Mice were injected with 70 kD FITC tagged dextran to compare the urinary excretion of a compound sieved similarly to albumin, based on size, but subject to different paths of tubular uptake and processing. As shown in Figure 1(d) total 24 hr urine excretion of 70 kDa dextran was 3-fold greater in OVE mice than in FVB mice. Fractionation of the urine on NAP5 columns (Figure 1(e)) or Sephadex G75 columns (Figure 1(f)) showed that almost all dextran in OVE and FVB urine was of high molecular weight. The 3-fold greater excretion of dextran in OVE, calculated from fluorescence in unfractionated urine, was consistent with calculations based on dextran in high molecular weight fractions from NAP5 or Sephadex columns.

### 3.2. Imaging TR-Albumin in Proximal Tubules

**Figure 2:**
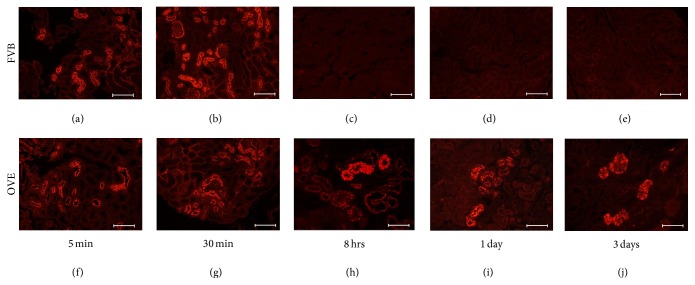
Time course to eliminate TR-albumin fluorescence in FVB and OVE kidneys. Tubule uptake and elimination of injected TR-albumin in FVB and OVE kidneys. Upper panels are from FVB and lower panels from OVE kidneys. Mice were sacrificed at the time points after TR-albumin injection indicated below the images. Initial uptake at 5 min or 30 min is similar in FVB (a, b) and OVE (f, g) tubules. The diabetic and FVB mice differ in that OVE tubules retain TR-albumin for 8 hrs, 1 day, and 3 days (h–j) while no FVB tubules retain TR-albumin at these time points (c–e). Images were made with a 20x objective and the bar in each panel equals 100 *μ*m.

Fluorescence from TR-albumin was used to visualize injected albumin in kidney sections. As shown in Figure 2 TR-albumin that leaked past the glomerulus was concentrated in tubules of OVE and FVB mice within 5 min after injection. Cells that took up TR-albumin were proximal tubule cells as indicated by the presence of megalin in all cells with albumin fluorescence (Supplemental Figure 1 in Supplementary Material available online at http://dx.doi.org/10.1155/2016/8749417) and the fact that TR-albumin fluorescence was observed in tubule cells contiguous with Bowman's capsule.

Proximal tubule fluorescence appeared to be similar in OVE and FVB tubules 5 min and 30 min after TR-albumin injection. However, marked differences in tubule fluorescence became obvious at 8 hours and continued 1 day and 3 days after injection. In all FVB tubules the injected albumin was cleared from tubules by 8 hours but in OVE tubules a subset retained strong fluorescence for days after injection. This was despite the fact that fluorescence remaining in serum had dropped to 4% of peak levels after 2 days in OVE and FVB mice. Many of the OVE tubules that retained TR-albumin were dilated and the epithelial cells were enlarged and engorged with albumin (Figures 2(h)–2(j)). Double staining for the endosome and lysosome marker lamp1 demonstrated partial colocalization (Supplemental Figure 2) with FITC tagged albumin 24 hours after injection. This pattern has been reported for lamp1 and endogenous albumin in normal mice [14].

### 3.3. Loss of Albumin Uptake in Tubules That Could Not Clear Albumin

**Figure 3:**
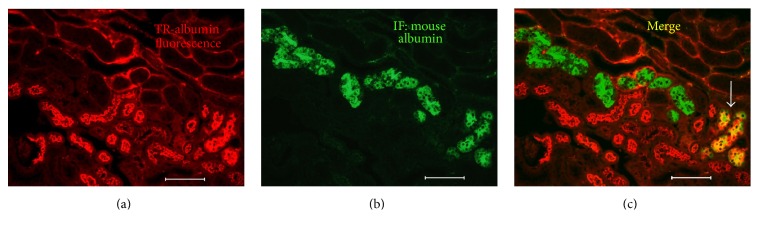
TR-albumin uptake and endogenous mouse albumin accumulation in OVE tubules. Most OVE tubules that take up TR-albumin, injected 5 min before sacrifice (image (a)), are not the same OVE tubules that accumulate endogenous mouse albumin, shown in image (b) by immunofluorescence (IF) staining for mouse albumin. In the merged image (c) only 3 tubule profiles are yellow indicating costaining for mouse albumin and TR-albumin (shown by the white arrow). Images were made with a 20x objective and the bar in each panel equals 100 *μ*m.

We previously reported that large amounts of mouse albumin accumulate in a subset of OVE proximal tubules [15]. To determine how accumulation of albumin in OVE proximal tubule cells affected subsequent protein reabsorption we compared uptake of TR-albumin to uptake of endogenous mouse albumin (Figure 3). Most OVE tubules that stained for accumulated endogenous mouse albumin (Figure 3(a)) did not take up injected TR-albumin (Figures 3(b) and 3(c)). Examination of 445 tubule cross-sections that stained strongly for mouse albumin from 4 OVE mice revealed that only 16% of these cross-sections showed uptake of injected TR-albumin. This suggests that prior accumulation of albumin impairs the ability of proximal tubule cells to take up TR-albumin.

**Figure 4:**
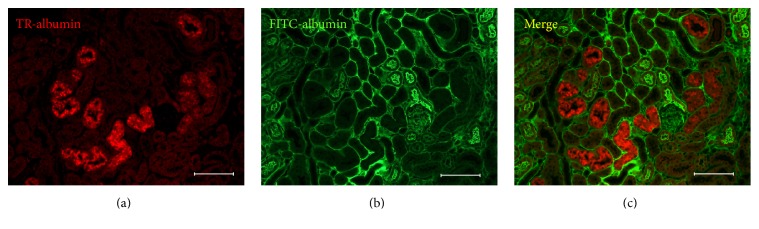
OVE tubule fluorescence for albumins injected 2 days apart. OVE tubules that retain TR-albumin fluorescence for two days after injection (panel (a)) do not take up FITC-albumin (panel (b)) injected, 5 min before sacrifice. In the merged image (panel (c)) no tubules are double stained. Images were made with a 20x objective and the bar in each panel equals 100 *μ*m.

**Figure 5:**
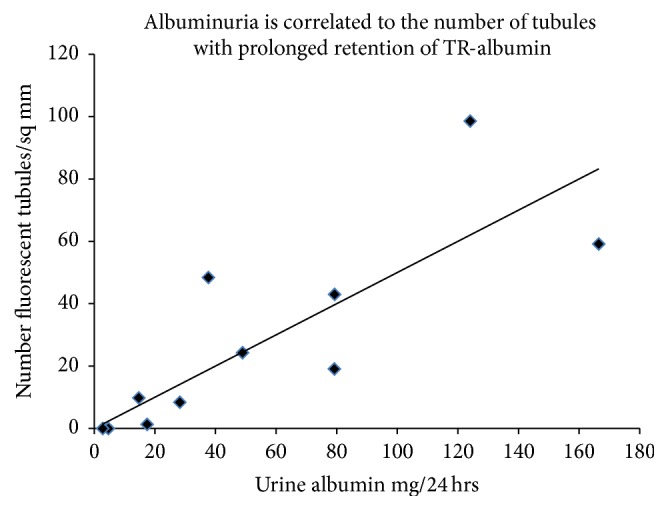
There is a significant correlation between albuminuria and the number of tubules with prolonged retention of TR-albumin. Each data point is from one OVE mouse and shows 24 hr urine albumin excretion of that mouse and the number of tubule segments counted with TR-albumin fluorescence per sq cm of kidney cortex 48 to 72 hours after the mouse was injected with TR-albumin. Pearson product correlation coefficient is 0.81, *P* ≤ 0.01 (*n* = 11). The trend line shows the best fit linear regression (slope = 0.48).

To estimate the time scale for loss of albumin uptake OVE mice were injected with fluorescent albumin 2 days apart: first with TR-albumin, followed 2 days later by a second injection of FITC tagged albumin (Figure 4). In these mice almost none of the tubules that retained fluorescence from the first TR-albumin injection (Figure 4(a)) stained for FITC-albumin from the second injection (Figures 4(b) and 4(c)). Therefore, tubules that had taken up and retained TR-albumin for 2 days were then unable to take up FITC-albumin. Since tubules with prolonged retention of TR-albumin were defective in their ability to take up additional albumin we examined whether TR-albumin retention was associated with the severity of albuminuria. As shown in Figure 5 the number of tubule segments that retained injected TR-albumin fluorescence for 2-3 days was significantly correlated with albuminuria.

### 3.4. Cell Death in Proximal Tubule Cells That Retain Albumin

**Figure 6:**
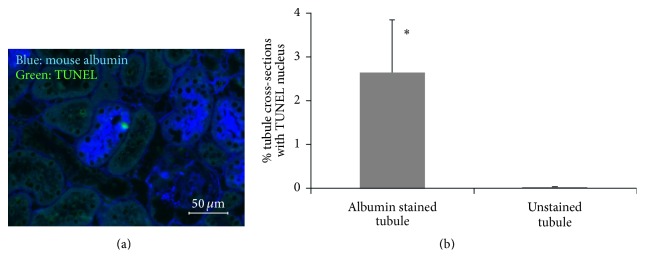
TUNEL stained cells are increased in OVE tubules that accumulate albumin. (a) Merged image of mouse albumin immunostaining (blue) and TUNEL staining (green) on an OVE section. (b) Quantitation of TUNEL staining in tubules with and without albumin staining from the same OVE kidney sections. Only TUNEL staining in tubular epithelial cells was counted. TUNEL stained cells within the tubule lumen or between tubules were not counted. Complete sections from 6 OVE mice were counted. *∗* indicates *P* < 0.05. TUNEL staining was performed as described in Section 2. The image was taken with a 40x objective and the bar indicates 50 *μ*m.

TUNEL staining was performed to determine if albumin accumulation increased the rate of tubule epithelial cell death (Figure 6). TUNEL staining in epithelial cells was rare in OVE tubules and even less frequent in FVB tubules (data not shown). In OVE tubules that accumulated albumin, the frequency of TUNEL staining was 76-fold greater than in OVE tubules with no albumin accumulation.

## 4. Discussion

The diabetic model with the most severe albuminuria is the OVE diabetic model [16] but the basis for the severity of proteinuria is unknown. There is a long standing question [10, 17] about the significance of impaired proximal tubule protein reuptake in diabetic albuminuria. Most studies have focused on defects in diabetic glomeruli, especially podocytes [13, 18]. Podocyte effacement, mutations in podocyte genes, or loss of podocytes results in increased glomerular permeability and leakage of albumin [19]. However Russo et al. [17] and Comper and Russo [20] have proposed that impaired tubular reabsorption of albumin is the primary cause of diabetic albuminuria. Our results in diabetic OVE mice show that both glomerular leakage and impaired tubular reabsorption contribute to the severity of OVE diabetic proteinuria.

Albumin and 70 kDa dextran have similar glomerular sieving coefficients [17] resulting in similar passage through the glomerular filtration barrier. Albumin is taken up by proximal tubule cells by specific mechanisms [14, 17] not used by dextran. If OVE proteinuria is due only to increased glomerular pore size, then leakage of dextran and albumin should be similarly affected. Conversely, if proteinuria is mostly due to impaired tubule uptake then leakage should be greater for albumin and leakage should be less for dextran. Results in Figure 1 demonstrate that diabetic urine contained at least 20-fold more injected bovine albumin than normal urine but only 3-fold more 70 kDa dextran. The greater impact of OVE diabetes on urine albumin compared to dextran suggested a more significant role for impaired tubule uptake. However, since output of both albumin and dextran was increased, then both increased glomerular leakage and impaired tubule uptake were implicated in OVE proteinuria.

Fluorescent tagged albumin provided a visual marker for tubule cell uptake and disposal of albumin. From 5 to 30 minutes after albumin injection there was no apparent difference between images of OVE and FVB mouse proximal tubule fluorescence (Figure 2). However after 8 hours the difference was obvious. By this time all FVB tubules and most OVE tubules were free of fluorescence. However a few OVE tubules retained strong TR-albumin fluorescence and continued to fluoresce for as long as 3 days after injection. Evidently, these long-fluorescing tubule cells could take up the injected TR-albumin but were unable to process and eliminate it. One day following injection of FITC tagged albumin, merged images of injected albumin and the protein lamp1, a marker for endosomes and lysosomes, demonstrated partial colocalization in OVE tubules (Supplemental Figure 2). The pattern was similar to what has been reported in normal tubules [14] and suggests that albumin was moving along normal processing pathways despite slow or interrupted processing. Proximal tubule cells apparently have a higher capacity to take up albumin than they can process. When OVE tubule cell processing capacity is exceeded they are unable to adequately suppress excessive uptake and accumulation of albumin continues. The most likely cause of too much proximal tubule cell uptake is very high concentrations of albumin in the tubule lumen from leaking glomeruli. We previously described structural abnormalities in the OVE glomerular filtration barrier [12, 13, 21–25] and functional results showing that a subset of OVE glomeruli have severe leakage of albumin [15]. Supplemental Figure 3 shows that OVE glomeruli still filter TR-albumin, despite possessing obviously abnormal podocyte organization, as revealed by a podocyte GFP transgene [26].

OVE mice were given consecutive injections of fluorescent albumin, two days apart. Tubules that retained fluorescence two days after the first albumin injection showed no fluorescence from the second albumin injection (Figure 4). This result indicates that tubules unable to fully process and eliminate internalized protein rapidly lose the ability to continue to take up albumin. We also found that albumin uptake was impaired in OVE tubules that were engorged with endogenous mouse albumin (Figure 3). Therefore experiments with injected fluorescent albumin and endogenous mouse albumin both showed that failure to clear internalized albumin interferes with subsequent albumin uptake. However, our methods were unable to determine if the tubules were permanently impaired. Conceivably some tubules may regain function. Permanently disabled tubules would lead to a progressive decline in the number of functional nephrons in the kidney, ultimately leading to renal failure. OVE tubules unable to take up protein provide an unobstructed pathway for intact albumin to pass into the urine. The significance of these abnormal tubules to total albuminuria is supported by the strong correlation (Figure 5) found between tubules unable to clear fluorescent albumin and the level of albuminuria in OVE mice.

The fact that some tubule cells were engorged with albumin, unable to clear internalized albumin, and impaired in albumin uptake indicated that these cells were not healthy and potentially predisposed to apoptosis. This was tested by TUNEL staining to identify dying cells. Within the same OVE kidney, tubule cell death was 76-fold higher if the tubule cells contained accumulated albumin than if they did not contain albumin (Figure 6). In a model of profound, nonselective proteinuria, Motoyoshi et al. [27] also found excessive apoptosis in tubule cells that accumulated cytoplasmic protein droplets. They attributed the increase in apoptosis to uptake of very high molecular protein, though their results could have been equally explained by intracellular protein accumulation. Tubular accumulation of albumin is found in biopsy specimens from human diabetics, which we have confirmed [15] in multiple diabetic biopsy specimens. OVE mice and advanced DN patients share similar pathologies of tubule albumin accumulation and urine excretion of large amounts of intact albumin [9].

## 5. Conclusions

Elevated urine output of injected dextran and greater output of injected albumin indicated that the severe albuminuria found in OVE diabetic mice was due to a combination of glomerular leakage and failure of tubules to reabsorb albumin. Visualization of fluorescent tagged albumin revealed a subset of OVE tubules unable to dispose of internalized albumin even after several days, which led to protein engorgement. This was associated with and probably a cause of impaired albumin uptake, increased albuminuria, and increased tubule cell death. Insufficient ability to process and dispose of internalized protein by proximal tubule cells promotes the progression of diabetic nephropathy.

## Supplementary Material

Supplementary material verifies additional points confirming conclusions about fluorescent albumin uptake and glomerular disruption in our diabetic model. Supplement Figure 1 shows that TR-albumin fluorescence is only in megalin containing tubule cells. Megalin is a marker of proximal tubules and demonstrates that normal uptake cells are used for injected albumin. Supplement Figure 2 demonstrates that FITC-albumin is partially localized with the endosome and lysosome marker protein lamp1 which is involved in normal processing of albumin. Supplement figure 3 uses a GFP marker in podocytes to show that fluorescent albumin is filtered even by structurally abnormal glomeruli in diabetic OVE mice.
