# Supplementary figures and images for: Impaired Albumin Uptake and Processing Promote Albuminuria in OVE26 Diabetic Mice
Source: J Diabetes Res. 2016 Oct 16;2016:8749417. doi: 10.1155/2016/8749417 (PMC5086391; doi:10.1155/2016/8749417)

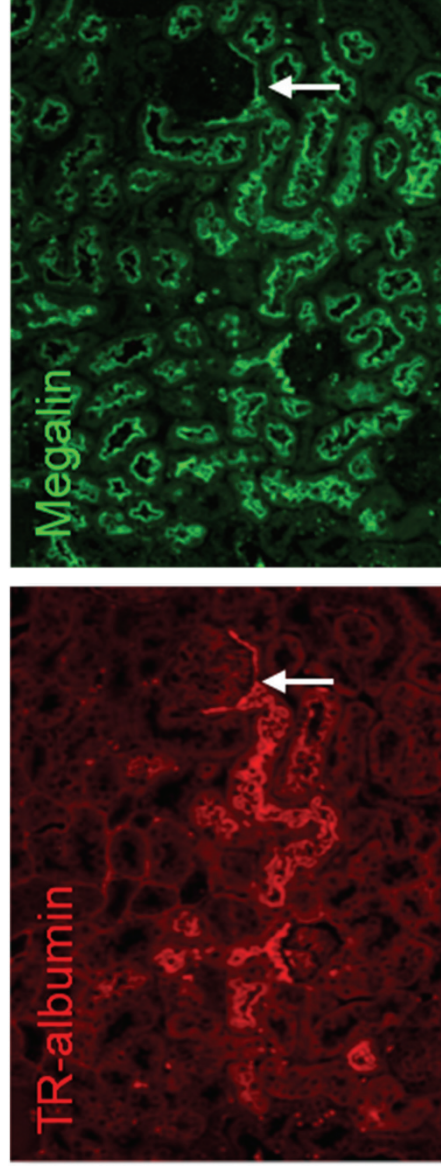

Supplement Figure 1

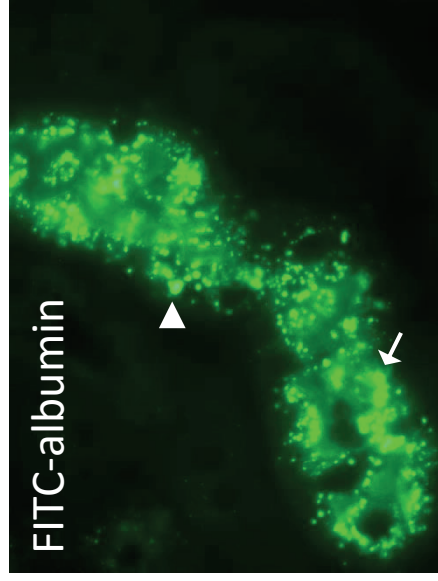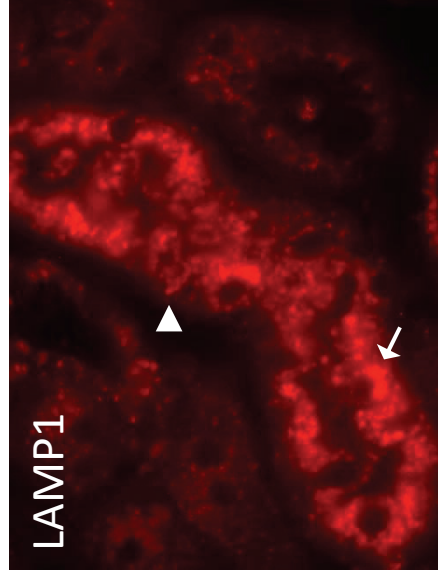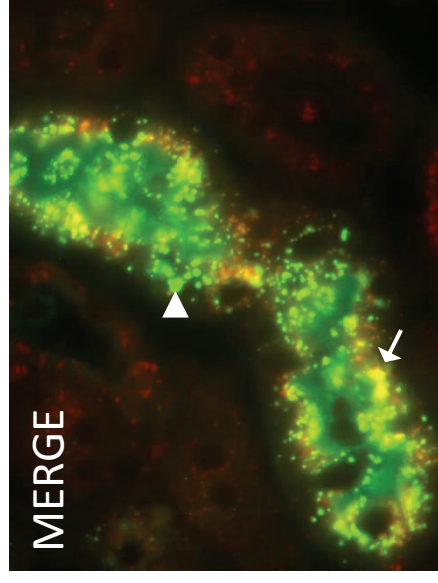

Supplement Figure 2

Supplement: Supplementary file 1 — Supplementary material verifies additional points confirming conclusions about fluorescent albumin uptake and glomerular disruption in our diabetic model. Supplement Figure 1 shows that TR-albumin fluorescence is only in megalin containing tubule cells. Megalin is a marker of proximal tubules and demonstrates that normal uptake cells are used for injected albumin. Supplement Figure 2 demonstrates that FITC-albumin is partially localized with the endosome and lysosome marker protein lamp1 which is involved in normal processing of albumin. Supplement figure 3 uses a GFP marker in podocytes to show that fluorescent albumin is filtered even by structurally abnormal glomeruli in diabetic OVE mice. [file 8749417.f1.pdf]
